# Relative telomer length as a potential biomarker in hepatocellular carcinoma: a comparative study of HCV patients treated with direct antiviral agent

**DOI:** 10.1186/s12885-025-15339-7

**Published:** 2026-02-23

**Authors:** Marwa Helal, Marwa Gamal, Ashraf A. Basuni, Walaa El Gendy, Ashraf Khalil

**Affiliations:** 1 Department of Biochemistry and Molecular Diagnostics, National Liver Institute, Menoufia University, Shebin Elkom, Egypt; 2 Department of Pathology, National Liver Institute, Menoufia University, Shebin Elkom, Egypt

**Keywords:** Hepatocellular carcinoma, Telomere length, Direct-acting antivirals, HCV, Molecular biomarkers

## Abstract

**Background:**

Hepatocellular carcinoma (HCC) remains a significant complication of hepatitis C virus (HCV) infection. This study investigates the potential of relative telomere length as a predictive biomarker in HCC patients, comparing non-DAA treatment groups with post-direct-acting antiviral (DAA).

**Methods:**

Telomere length was measured using quantitative real-time PCR (qPCR) in tumor and adjacent non-tumorous tissues from 41 HCC patients, divided into de novo (*n* = 16) and post-DAA (*n* = 25) groups.

**Results:**

The mean telomere length was significantly higher in tumor than non-tumorous tissues (3.39 ± 4.05 compared to 1.01 ± 0.04; *p* = 0.001). Mean telomere length varied significantly between groups, with non-DAA group showing higher tumor telomere length (5.15 ± 4.88) compared to post-DAA patients (2.26 ± 3.01). Receiver Operating Characteristic (ROC) curve analysis revealed fair discriminatory ability in the non-DAA group (AUC 0.706, 95% CI: 0.524–0.888, *p* = 0.047). A significant correlation between tumor telomere length and carcinoembryonic antigen was observed in the post-DAA group. Non-DAA group showed more aggressive tumor grades, while post-DAA patients had better liver function. No significant association was found between relative telomere length and HCC risk.

**Conclusion:**

Significant telomere length alterations and clinicopathological differences highlight molecular heterogeneity in HCV-related HCC, particularly post-DAA. While Relative telomere length (RTL) did not predict HCC risk, its correlations with tumor markers suggest potential as a prognostic biomarker, warranting further research in larger cohorts.

## Introduction

Hepatocellular carcinoma (HCC) represents a critical global health challenge, with liver cancer ranking as the sixth most common cancer and the third leading cause of cancer-related mortality worldwide [1]. The complex etiology of HCC is predominantly associated with chronic viral hepatitis, particularly HCV infection, which remains a significant risk factor for liver disease progression and hepatocarcinogenesis [2, 3]. The molecular mechanisms underlying HCC are intricate and multifaceted, involving a complex interplay of genetic, environmental, and viral factors [4–6]. Chronic viral infection triggers persistent inflammation, oxidative stress, and progressive liver damage, creating a pro-carcinogenic microenvironment [7]. At the cellular level, these processes lead to significant molecular alterations, with telomere biology emerging as a crucial area of investigation in understanding cancer development and progression [8, 9]. Telomeres, the protective chromosomal end caps, play a fundamental role in maintaining genomic stability and cellular senescence [10]. In healthy hepatocytes, telomeres undergo progressive shortening during chronic liver injury and inflammation, contributing to cellular aging and potential malignant transformation [11, 12]. The liver, typically a quiescent organ, experiences complex telomere dynamics during chronic disease progression, with telomerase activity becoming a critical molecular switch in hepatocarcinogenesis [13, 14]. The molecular landscape of telomere maintenance in HCC is particularly prominent. Telomerase reactivation is observed in over 90% of HCC cases, primarily through telomerase promoter mutations, viral insertions, or chromosomal rearrangements [15–18]. This reactivation represents a key event in early liver tumor development, enabling cancer cells to overcome replicative senescence and achieve indefinite proliferative potential [19, 20].

Recent advances in hepatitis C treatment, particularly the introduction of direct-acting antiviral agents (DAA), have revolutionized viral hepatitis management [3, 4]. These innovative treatments offer unprecedented viral clearance rates, with sustained virologic response exceeding 90%. However, the long-term molecular consequences of these treatments on liver disease progression remain incompletely understood [21, 22]. Also, subsequent research revealed potential complexities, with concerns emerging about unexpected molecular interactions and possible increases in HCC incidence following DAA therapy. These findings underscore the need for comprehensive investigations into the long-term molecular consequences of antiviral treatments [23–25].

The relationship between viral clearance, telomere dynamics, and HCC risk presents a complex and evolving research landscape. Emerging evidence suggests that viral hepatitis and chronic inflammation significantly influence telomere length, potentially modifying cancer risk and progression [26, 27]. Multiple studies have demonstrated the critical role of telomere length as a potential biomarker in various malignancies, with liver disease presenting a particularly intriguing molecular environment [26–29]. Several key molecular mechanisms underscore the importance of telomere research in HCC: telomere shortening contributes to chromosomal instability, telomerase reactivation enables cancer cell immortalization, chronic inflammation accelerates telomere erosion, and treatment histories may modify molecular risk factors [26–29].

This study investigated telomere length in tumor and adjacent non-tumorous liver tissues of HCC patients with chronic HCV, comparing DAA-treated and DAA-naive groups, to evaluate its potential as a prognostic biomarker for HCC development following DAA therapy.

## Patients and methods

This study was conducted at the National Liver Institute, Menoufia University, Egypt, in the Departments of Clinical Biochemistry and Pathology from March 2023 to March 2024. It was approved by the ethics committee (IRB 00014014/FWA00034015), with written consent obtained from all participants. All patients had confirmed chronic HCV infection (via serology and PCR) and evidence of cirrhosis (100% prevalence across fibrosis stages F1-F4, as detailed in Table 1), a key high-risk factor for HCC. Paraffin-embedded tissue sections were collected from HCC patients with chronic HCV infection, divided into two groups: Post-DAA (*n* = 25, sofosbuvir-based regimens achieving sustained virologic response [SVR] after standard 12-week treatment; duration 11–13 weeks) and Non-DAA (*n* = 16, treatment-naïve). HCC diagnosis was confirmed via histopathological examination. Tumor sections contained >80% tumor cells, and adjacent non-tumorous (ANT) liver tissue sections >90% non-tumor cells, verified by H&E staining by a blinded pathologist to minimize cellular heterogeneity effects. Paired tumor and adjacent non-tumorous (ANT) liver tissue samples were obtained, with five 10-micron sections per tissue type for DNA extraction and telomere length analysis. Tumor staging used the TNM Classification (8th Edition) [30], and differentiation was assessed with the Edmondson-Steiner grading system. Clinical and demographic data, including HCV history and DAA treatment details, were retrospectively collected from medical records. Viral load data pre-DAA were not uniformly available due to retrospective design; this is noted as a limitation.

**Table 1:** The demographic and the clinicopathological features in the non-DAA and post-DAA groups

	Category	Non-DAA (*n* = 16)	Post-DAA (*n* = 25)	*X*^2^	*p*-value
Age	Mean ± SD	59.75 ± 10.28	59.40 ± 10.84	0.10	0.075
Gender	Female	5 (31.3%)	2 (8%)	0.054	0.089
	Male	11 (68.7%)	23 (92%)		
Liver Size	Enlarged	4 (25%)	6 (24%)	0.942	1.000
	Normal	12 (75%)	19 (76%)		
Spleen Size	Enlarged	8 (50.0%)	8 (32%)	0.249	0.330
	Normal	8 (50.0%)	17 (68%)		
FLN	Multiple	6 (37.5%)	10 (40%)	0.873	1.000
	Solitary	10 (62.5%)	15 (60%)		
Histological Grade	GII	6 (37.5%)	18 (72%)	0.029	0.050
	GIII	10 (62.5%)	7 (28%)		
LVI	Absent	3 (18.8%)	8 (32%)	0.350	0.478
	Present	13 (81.2%)	17 (68%)		
PNI	Absent	16 (100%)	24 (96%)	0.418	1.000
	Present	0 (0.0%)	1 (4%)		
Pathological T-Stage					
Early stage	T1a/T1b	2 (12.5%)	6 (24.0%)	0.533	0.601
Intermediate stage	T2	8 (50.0%)	12 (48.0%)	0.001	0.988
Advanced stage	T3/T4	6 (37.5%)	7 (28.0%)	0.325	0.519
Fibrosis Stage	F1	4 (25.0%)	10 (40%)	0.069	-
	F2	7 (43.8%)	3 (12%)		
	F4	5 (31.2%)	12 (48%)		
Child-Pugh Score	A5	2 (12.5%)	16 (64%)	0.001	-
	A6	4 (25.0%)	4 (16%)		
	B	10 (62.5%)	3 (12%)		
	C	0 (0.0%)	2 (8%)		

*HCC* Hepatocellular carcinoma, *DAA *Direct-acting antiviral, *Post-DAA *HCC developed after post direct antiviral therapy for HCV, *Non-DAA *HCC did not receive prior DAA for HCV, *FLN *Focal lesion number, *LVI* Lymphovascular invasion, *PNI *Perineural invasion, *HBsAb *Hepatitis B surface antibody, *HIV Ab *Human Immunodeficiency Virus antibody, *HCV Ab *HCV antibody, *Child-Pugh *A scoring system used to assess the prognosis of chronic liver disease

### DNA extraction and telomere length quantification

Genomic DNA was extracted from formalin-fixed paraffin-embedded (FFPE) tissue samples using the QIAamp DNA FFPE Tissue Kit (QIAGEN, Hilden, Germany), following the manufacturer’s recommended protocol. DNA quality and concentration were rigorously assessed using a Thermo Scientific NanoDrop spectrophotometer, with quality control parameters including DNA yield measurement, 260/280 nm and 260/230 nm ratio purity assessment (ratios > 1.8 and > 2.0, respectively), and verification of DNA integrity. All extractions were performed in triplicate to ensure reproducibility. Telomere length was quantified using quantitative real-time PCR (qRT-PCR) on a Real-Time 7500 Fast system (Applied Biosystems, Foster City, CA, USA). The experimental design utilized specialized primer sets for telomere repeat sequences: Telomere Repeat Primers:

Forward:5’-CGGTTTGTTTGGGTTTGGGTTTGGGTTTGGGTTTGGGTT-3’. Reverse: 5’-GGCTTGCCTTACCCTTACCCTTACCCTTACCCTTACCCT-3’. The single-Copy Gene (Reference Gene) Primers: Forward: 5’-CAGCAAGTGGGAAGGTGTAATCC-3’. Reverse: 5’-CCCATTCTATCATCAACGGGTACAA-3’. The single-copy housekeeping gene 36B4 (acidic ribosomal phosphoprotein P0) served as the reference to normalize for inter-sample DNA input variations in quantity, quality, and extraction efficiency, enabling reliable RTL quantification as the ratio of telomere repeat copies to single-copy gene copies per diploid genome. Each 20 µL reaction mixture was carefully composed to include 10 µL Maxima SYBR Green qPCR Master Mix, 1 µL each of forward and reverse primers, 6 µL nuclease-free water, and 2 µL template DNA. The thermal cycling protocol began with an initial denaturation at 95 °C for 3 min, followed by 40 amplification cycles of 15 s at 95 °C and 1 min at 56 °C. Amplification efficiency was validated at 95–105% using serial dilutions for standard curves (*R*² >0.99). Relative telomere length (RTL) was calculated using the 2-ΔΔCt method, comparing cycle threshold values of telomere repeat copy number to the single-copy gene reference.

### Statistical analysis

Statistical analyses were conducted using SPSS version 25.0 (IBM Corp., Armonk, NY, USA). RTL differences between the Non-DAA HCC Group and Post-DAA HCC Group were evaluated using independent samples t-tests, assuming unequal variances due to preliminary assessments. Normality of telomere length data was confirmed through prior testing, supporting the use of parametric methods. Pearson correlation analyses and multivariate statistical approaches were also applied to explore relationships and adjust for relevant covariates. Receiver operating characteristic (ROC) curves were generated to assess the discriminatory ability of RTL, with optimal cutoff values determined using Youden’s Index (sensitivity + specificity − 1) to maximize the balance between sensitivity and specificity. The t-test was selected for its ability to compare means and provide confidence intervals for the mean difference, facilitating interpretation of biological significance in telomere length variations. Multivariate models adjusted for key confounders including age, fibrosis stage, and Child-Pugh score. Statistical significance was defined as *p* < 0.05.

## Results

### Clinicopathological characteristics

Table 1 presents a comprehensive comparative analysis of clinicopathological features between non-DAA and post-DAA HCC patients. Patient demographics revealed comparable mean ages (non-DAA: 59.75 ± 10.28 years; post-DAA: 59.40 ± 10.84 years; *p* = 0.075), with a notable gender disparity characterized by a higher proportion of males in the post-DAA group (92% vs. 68.7%), though this difference did not reach statistical significance (χ² = 0.054, *p* = 0.089).

Histopathological analysis emerged as a critical distinguishing feature, demonstrating a statistically significant difference in tumor grading (χ²=0.029, *p* = 0.05). The non-DAA group exhibited a predominance of Grade III tumors (62.5%) compared to the post-DAA cohort (28.0%). Pathological T-stage distribution showed no statistically significant variations, with early stage tumors observed in 12.5% of de novo and 24.0% of post-DAA patients, intermediate stage tumors in 50.0% and 48.0%, and advanced stage tumors in 37.5% and 28.0%, respectively.

Subsidiary hepatic characteristics demonstrated minimal intergroup differences. Liver size and focal liver number (FLN) distribution showed no statistically significant variations (*p* = 1.00). Lymphovascular invasion (LVI) was present in 81.2% of non-DAA cases and 68% of post-DAA patients, with no significant statistical difference. Perineural invasion was rare, with only one patient in the post-DAA group demonstrating this feature. Liver functional assessment revealed profound differences in Child-Pugh classification (*p* = 0.001), with the post-DAA group predominantly classified as A5 (64%), in stark contrast to the non-DAA group’s predominantly Class B classification (62.5%). Fibrosis staging approached statistical significance (*p* = 0.069), with post-DAA patients more frequently presenting advanced fibrosis (F4).

### Biochemical and hematological profiles

Table 2 displayed a comparative analysis of the biochemical and hematological parameters between patients in the P-DAA and non-DAA groups. Biochemical, renal, and tumor marker profiles were generally comparable between the post-DAA and non-DAA groups. No significant differences were observed in liver enzymes (AST, ALT, ALP), bilirubin levels, total protein, albumin, or inflammatory markers such as CRP and LDH. Similarly, renal function markers (urea and creatinine) showed no statistically significant variation. However, gamma glutamyl transferase (GGT) was significantly elevated in the non-DAA group (*p* = 0.038), indicating a possible difference in cholestatic injury patterns. Regarding tumor markers, alpha-fetoprotein (AFP) and CA19.9 levels were not significantly different, whereas carcinoembryonic antigen (CEA) was significantly higher in the non-DAA group (*p* = 0.017).

**Table 2:** Comparison of biochemical, and hematological profiles between post-DAA and non-DAA groups

	Post-DAA (*n* = 25)	Non-DAA (*n* = 16)	
Parameter	Mean ± SD	Mean ± SD	*p*-value
Total Bilirubin (mg/dL)	1.27 ± 1.41	2.31 ± 4.01	0.328
Direct Bilirubin (mg/dL)	0.63 ± 0.80	0.51 ± 0.43	0.547
Total Protein (g/dL)	6.82 ± 1.43	6.58 ± 1.67	0.635
Albumin (g/dL)	3.71 ± 0.88	3.29 ± 0.90	0.153
AST (U/L)	116.80 ± 167.78	133.75 ± 165.49	0.752
ALT (U/L)	129.28 ± 196.24	103.31 ± 122.53	0.605
ALK. Phosphatase (U/L)	100.28 ± 59.28	124.85 ± 98.21	0.377
GGT (U/L)	67.65 ± 89.80	145.12 ± 122.17	0.038
Urea (mg/dL)	46.20 ± 50.43	39.56 ± 15.87	0.545
Creatinine (mg/dL)	1.01 ± 0.72	6.39 ± 21.24	0.327
CRP (mg/L)	19.12 ± 23.09	23.75 ± 36.62	0.656
LDH (U/L)	334.60 ± 205.04	455.81 ± 366.27	0.240
Lactate	26.07 ± 17.89	29.67 ± 11.73	0.493
AFP	596 ± 2046	3215 ± 7972	0.124
CA19.9	73.73 ± 116.69	500 ± 1932	0.274
CEA	1.87 ± 0.87	2.65 ± 1.13	0.017
INR IU	1.34 ± 0.37	1.37 ± 0.71	0.854
PT/Sec	16.40 ± 6.87	13.57 ± 4.95	0.162
PT conc. %	0.63 ± 0.23	0.75 ± 0.22	0.096
Hb g/dL	12.28 ± 2.37	12.18 ± 1.90	0.882
Ht%	35.66 ± 6.99	37.33 ± 15.49	0.642
WBCs10^6^/l	9.73 ± 8.06	9.69 ± 6.99	0.989
Platelets10^9^/l	191.60 ± 78.90	170.63 ± 92.08	0.441

Comparisons were conducted using the independent samples t-test without assuming equal variances (Welch's t-test), due to the results of Levene’s test for equality of variances. A *p*-value less than 0.05 was considered statistically significant

*SD* standard deviation, *P-DAA* HCC developed after post direct antiviral therapy for HCV, *Non-DAA* HCC did not receive prior DAA for HCV. Equal Variance Not Assumed (Welch's t-test), *DB* Direct Bilirubin, *TB *Total Bilirubin, *TP *Total Protein, *ALB *Albumin, *P *Phosphorus, *ALP *Alkaline Phosphatase, *GGT *Gamma Glutamyl Transferase, *AST *Aspartate Aminotransferase, *ALT *Alanine Aminotransferase, *SD *standard deviation, *P-DAA *HCC developed after post direct antiviral therapy for HCV, *De novo *HCC did not receive prior DAA for HCV, *Hb *hemoglobin, *Ht *Hematocrit, *WBCs *White Blood Cells, *PT *prothrombin time, *INR *International Normalized Ratio

Comparison of hematological and coagulation profiles between the post-DAA and Non-DAA groups revealed no statistically significant differences across the assessed parameters and were all comparable between groups all *p* > 0.05.

### Relative telomere length in tumor and non-tumorous tissues

Table 3 presents a comprehensive analysis of RTL in tumorous and ANT liver tissues in HCC patients, both overall and stratified by non-DAA and post-DAA treatment groups. A paired samples t-test revealed statistically significant differences in telomere length between tumorous and non-tumorous tissues across all groups. In the overall HCC cohort (*n* = 41), the mean telomere length in tumorous tissues was significantly elevated at 3.39 ± 4.05 compared to 1.01 ± 0.04 in non-tumorous tissues (t = 3.757, *p* = 0.001), with a mean paired difference of 2.38 ± 4.06. A weak, non-significant negative correlation was observed between Tlm-Tu and Tlm-NonTu (*r*=−0.141, *p* = 0.379), suggesting tumor-specific alterations in telomere maintenance mechanisms. Non-tumorous tissue telomere lengths clustered tightly around 1.00, while tumorous tissue lengths exhibited greater variability.

**Table 3:** Relative telomere length in HCC tumor and adjacent non-tumorous tissues across patient groups

Group	RTL	Mean ± SD	Paired Difference Tlm (Tu –NonTu)	95% CI of Difference (Lower, Upper)	t-value	*p*-value	Pearson *r*	Correlation *p*-value
Overall HCC (*n* = 41)	Tu	3.39 ± 4.05	2.38 ± 4.06	1.10, 3.66	3.757	0.001**	−0.141	0.379
	NonTu	1.01 ± 0.04						
Non-DAA (*n* = 16)	Tu	5.15 ± 4.88	4.14 ± 4.89	1.53, 6.74	3.383	0.004**	−0.267	0.318
	NonTu	1.02 ± 0.06						
Post-DAA (*n* = 25)	Tu	2.26 ± 3.01	1.25 ± 3.01	0.01, 2.49	2.082	0.048*	−0.145	0.490
	NonTu	1.00 ± 0.01						

*HCC* Hepatocellular carcinoma, *RTL* Relative telomere length, *Tu* Tumor, *NonTu* non-tumorous tissue, *SD* Standard deviation, *Non-DAA* HCC patients who did not receive prior direct-acting antiviral (DAA) therapy for HCV, *Post-DAA* HCC patients who developed HCC after DAA therapy for HCV

Statistical significance: *p < 0.05, **p < 0.01

In the non-DAA group (*n* = 16), the difference was more pronounced, with Tlm-Tu at 5.15 ± 4.88 compared to Tlm-NonTu at 1.02 ± 0.06, yielding a mean paired difference of 4.14 ± 4.89 (t = 3.383, *p* = 0.004). The correlation remained weak and non-significant (*r* =−0.267, *p* = 0.318). In the post-DAA group (*n* = 25), a more modest elevation was observed, with Tlm-Tu at 2.26 ± 3.01 and Tlm-NonTu at 1.00 ± 0.01, resulting in a mean paired difference of 1.25 ± 3.01 (t = 2.082, *p* = 0.048). The correlation was similarly weak and non-significant (*r* =−0.145, *p* = 0.490). These findings highlight a consistent pattern of elevated telomere length in tumorous tissues across HCC patients, with greater variability in the non-DAA group, potentially reflecting distinct telomere dynamics influenced by prior DAA therapy. The tight clustering of non-tumorous tissue values suggests stable telomere maintenance in ANT tissues, while the variability in tumorous tissues may indicate heterogeneous tumor biology.

### Correlations of relative telomere length with tumor markers

Table 4 presents the correlation analysis of telomere length in the tumorous and non-tumorous tissue, as well as with tumor markers in non-DAA and post-DAA HCC groups. In the non-DAA group RTL in the tumorous tissue showed weak, statistically non-significant correlations with non-tumorous RTL (*r* = 0.667, *p* = 0.318), however, in the post-DAA group it showed weak non-significant negative correlation (*r*= −0.145, *p* = 0.490).

**Table 4:** Correlation of RTL with tumor markers, in non-DAA and P-DAA groups

Group	Non-DAA (*n* = 16)				Post-DAA (*n* = 25)			
	RTL-NonTu		RTL-Tu		RTL-NonTu		RTL-Tu	
Correlations	*r*	*p*	*r*	*p*	*r*	*p*	*r*	*p*
RTL-NonTu	1.000	−0.00	0.267	0.318	1.000	−0.00	−0.145	0.490
RTL-Tu	0.267	0.318	1.000	−0.00	−0.145	0.490	1.000	−0.00
AFP	0.106	0.695	0.301	0.257	−0.058	0.782	−0.142	0.497
CA19.9	−0.069	0.799	−0.143	0.598	0.268	0.195	−0.176	0.401
CEA	−0.298	0.262	0.293	0.270	0.101	0.631	0.463	*0.020

*Post-DAA* HCC developed after post direct antiviral therapy for HCV, *Non-DAA* HCC did not receive prior DAA for HCV, *RTL-Tu* Relative telomere length in tumor tissue, *Tel-NonTu* Relative telomere length in non-tumor tissue, *AFP* Alpha-fetoprotein, *CA19.9* Carbohydrate Antigen 19-9, *CEA* Carcinoembryonic Antigen, *r* Pearson correlation coefficient, *p* = significance (2-tailed)

* Indicates statistical significance at the 0.05 level

Correlation analysis of telomere length with tumor markers AFP, CEA and CA19-9 revealed: In the non-DAA group, RTL showed weak, statistically non-significant correlations with tumor markers (all *p* > 0.05). AFP displayed a positive correlation with tumor telomere length (*r* = 0.301, *p* = 0.257), while CEA exhibited a similar weak positive correlation (*r* = 0.293, *p* = 0.270). CA19-9 showed negative correlation (*r*=−0.143, *p* = 0.598). Non-tumorous RTL demonstrated weak positive correlation with AFP (*r* = 0.106, *p* = 0.695) and weak negative correlations with CEA (*r* =−0.298, *p* = 0.262), and CA19-9 (*r*=−0.690 *p* = 0.799). The post-DAA group revealed a distinctly different correlation pattern. A statistically significant positive correlation emerged between tumor RTL and CEA (*r* = 0.463, *p* = 0.020), representing the most notable finding in this group. Other tumor markers AFP and CA19-9 showed weak non-significant negative associations with tumor telomere length. Non-tumorous RTL did not show any significant correlation with all tumor marker all *p* > 0.05.

### ROC curve analysis of relative telomere length

**Fig. 1:**
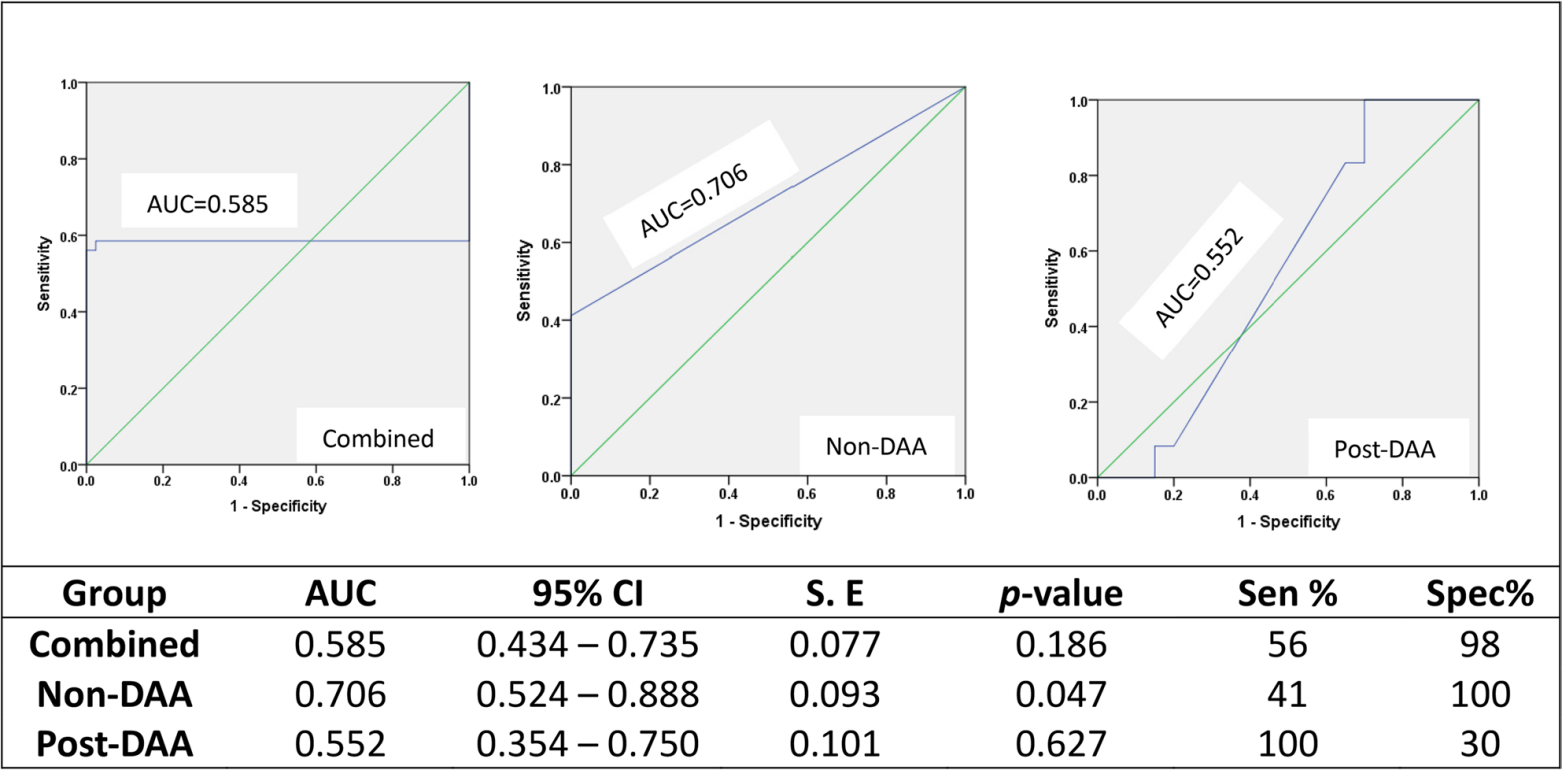
Receiver operating characteristic (ROC) curves for RTL in ANT liver tissue to discriminate paired HCC tumor from non-tumor tissue. Curves represent the combined cohort (*n*=41) and subgroups: Non-DAA (*n*=20) and Post-DAA (*n*=21). Optimal cutoffs were determined using Youden's Index (sensitivity + specificity - 1). Area under the curve (AUC) values with 95% confidence intervals (CI) and *p*-values are shown for each curve. Combined: AUC = 0.585 (95% CI: 0.434–0.735, *p* = 0.186); optimal cutoff = 1.12 (sensitivity = 59%, specificity = 98%). Non-DAA: AUC = 0.706 (95% CI: 0.524–0.888, *p* = 0.047); optimal cutoff = 0.50 (sensitivity = 41%, specificity = 100%). Post-DAA: AUC= 0.552 (95% CI: 0.354–0.750, *p* = 0.627); optimal cutoff = 1.00 (sensitivity = 100%, specificity = 30%). Post-DAA: HCC developed after post direct antiviral therapy for HCV. Non-DAA: HCC did not receive prior DAA for HCV, Combined: Non-DAA+ Post-DAA), AUC area under the curve, CI: confidence interval, S.E:Standard Error, Sen: sensitivity, Spec: Specificity, Statistical significance: *p* < 0.05

ROC Curve Analysis of Relative Telomere Length Fig. 1 displays the ROC curves evaluating the discriminatory performance of RTL measured in adjacent non-tumorous (ANT) liver tissue to distinguish paired HCC tumor from non-tumor states within each group. Curves are shown for the overall cohort and subgroups, with key performance metrics as follows: Combined cohort (*n* = 41; non-DAA + post-DAA HCC vs. corresponding non-tumorous tissue): AUC = 0.585 (95% CI: 0.434–0.735, *p* = 0.186), indicating poor and non-significant discriminatory performance ~ at optimal cutoff of 1.12 (sensitivity = 56%, specificity = 98%). Non-DAA group (*n* = 16): AUC = 0.706 (95% CI: 0.524–0.888, *p* = 0.047), demonstrating fair discriminatory ability (sensitivity = 41%, specificity = 100% at optimal cutoff of 0.50), suggesting potential utility of non-tumorous RTL in predicting malignant transformation among untreated HCV patients despite limited sample size and data ties. Post-DAA group (*n* = 25): AUC = 0.552 (95% CI: 0.354–0.750, *p* = 0.627), reflecting limited and non-significant predictive value ~ at optimal cutoff of 1.00 (sensitivity = 100%, specificity = 30%).

### Multivariate logistic regression and association with HCC risk

Table 5 integrates multivariate logistic regression analysis of RTL with clinical-pathological features and the association of RTL with HCC risk in non-DAA (*n* = 16) and post-DAA (*n* = 25) patient groups, compared to a synthetic healthy control group (*n* = 41) derived from ANT tissue telomere length values (mean RTL = 1.00). In the multivariate logistic regression analysis, distinct molecular interactions between RTL and clinical-pathological features were observed between the non-DAA and post-DAA groups. In the non-DAA group, estimated tumor volume showed a strong negative correlation with RTL (coefficient: −2.251, OR: 0.105), indicating that shorter telomere lengths were associated with larger tumor volumes. Histological grade exhibited a notable positive correlation (coefficient: 0.748, OR: 2.112), suggesting that longer telomere lengths were linked to more aggressive tumor characteristics. Age showed a moderate positive association (coefficient: 0.351, OR: 1.421), while fibrosis stage and Child-Pugh classification had minimal impact (ORs close to 1). In contrast, the post-DAA group displayed different dynamics: estimated tumor volume was positively correlated with RTL (coefficient: 0.585, OR: 1.794), suggesting treatment-induced alterations in tumor biology. Child-Pugh classification showed a significant positive association (coefficient: 0.532, OR: 1.702), indicating a link between RTL and liver functional status post-DAA therapy. Histological grade had a modest positive correlation (coefficient: 0.265, OR: 1.304), and fibrosis stage showed a negative association (coefficient: −0.642, OR: 0.526).

**Table 5:** Multivariate logistic regression and association of RTL with HCC risk in non-DAA and post-DAA groups

Analysis Type	Parameter	Non-DAA (*n* = 16)	Post-DAA (*n* = 25)	Adjusted OR [95% CI]	*p*-value
Multivariate Logistic Regression: Clinical-Pathological Features					
Numerical Features					
	Age (Coefficient, OR)	0.351, 1.421	−0.538, 0.584	-	-
	Estimated Tumor Volume (Coefficient, OR)	−2.251, 0.105	0.585, 1.794	-	-
Categorical Features					
	Histological Grade (Coefficient, OR)	0.748, 2.112	0.265, 1.304	-	-
	Pathological Staging (Coefficient, OR)	0, 1.000	0, 1.000	-	-
	Fibrosis Stage (Coefficient, OR)	0.079, 1.082	−0.642, 0.526	-	-
	Child-Pugh Classification (Coefficient, OR)	0.065, 1.067	0.532, 1.702	-	-
Association with HCC Risk					
Telomere Length (Based on Mean)					
	Short (0.01–1.00)	8 (50.0%)	17 (68.0%)	1.00 (Reference)	-
	Long (1.00–15.50)	8 (50.0%)	8 (32.0%)	0.81 [0.37–1.77]	0.595
Telomere Length by Quartiles					
	First Quartile (0.01–0.37)	5 (31.2%)	7 (28.0%)	1.00 (Reference)	-
	Second Quartile (0.37–1.76)	2 (12.5%)	8 (32.0%)	1.00 [0.39–2.57]	0.996
	Third Quartile (1.76–4.25)	4 (25.0%)	6 (24.0%)	0.90 [0.34–2.38]	0.827
	Fourth Quartile (4.25–15.50)	5 (31.2%)	4 (16.0%)	0.62 [0.22–1.75]	0.367
Group Characteristics					
	Mean Telomere Length	5.12 ± 4.70	2.43 ± 3.10	-	-
	Median Telomere Length	2.38	0.87	-	-
	P for Trend (Quartiles)	-	-	-	0.177

*RTL* Relative telomere length, *OR *Odds ratio, *CI *Confidence interval, *Non-DAA *HCC patients who did not receive prior direct-acting antiviral (DAA) therapy for HCV, *Post-DAA *HCC patients who developed HCC after DAA therapy for HCV. The multivariate logistic regression analysis examines the relationship between RTL and clinical-pathological features. The HCC risk analysis compares RTL in non-DAA and post-DAA groups to a synthetic healthy control group (*n*=41) derived from ANT tissue telomere length values (mean RTL = 1.00). RTL was dichotomized into Short (0.01–1.00) and Long (1.00–15.50) based on the mean RTL of healthy controls and categorized into quartiles (Q1: 0.01–0.37, Q2: 0.37–1.76, Q3: 1.76–4.25, Q4: 4.25–15.50)

For HCC risk analysis, RTL was dichotomized into Short (0.01–1.00) and Long (1.00–15.50) based on the mean RTL of healthy controls and categorized into quartiles (Q1: 0.01–0.37, Q2: 0.37–1.76, Q3: 1.76–4.25, Q4: 4.25–15.50). The adjusted odds ratio for Long vs. Short RTL was 0.81 (95% CI: 0.37–1.77, *p* = 0.595), suggesting a reduced HCC risk with longer telomeres, though not statistically significant. Quartile analysis showed ORs of 1.00 (95% CI: 0.39–2.57, *p* = 0.996) for Q2, 0.90 (95% CI: 0.34–2.38, *p* = 0.827) for Q3, and 0.62 (95% CI: 0.22–1.75, *p* = 0.367) for Q4, compared to Q1, with a non-significant *p*-value for trend (*p* = 0.177), indicating no clear dose-response relationship. The non-DAA group had a higher mean RTL (5.12 ± 4.70) and median RTL (2.38) compared to the post-DAA group (mean: 2.43 ± 3.10, median: 0.87), reflecting potential differences in telomere dynamics influenced by DAA therapy.

## Discussion

This study uncovered significant molecular and clinicopathological insights into HCC among de novo and post-DAA groups in chronic HCV patients, highlighting telomere length as a dynamic marker of tumor biology. A key finding was the significant increase in the RTL in tumorous tissue compared to ANT tissue in both groups, with greater elongation in the non-DAA group, suggesting tumor-specific telomere maintenance alterations. Additionally, a significant correlation between tumorous RTL and CEA in the post-DAA group points to DAA-induced molecular adaptations, potentially linking telomere dynamics to broader gastrointestinal carcinogenesis. Clinicopathologically, the non-DAA group showed more aggressive tumor grades, while the post-DAA group had better liver function, indicating DAA therapy’s influence on HCC presentation. These findings underscore RTL potential as a marker for HCC heterogeneity and personalized risk assessment [31]. The significant increase in RTL in tumorous tissue aligns with prior research. Feng et al. 2017, reported similar RTL elongation in HCC tumor tissue, suggesting telomere length reflects disease persistence and tumor-specific molecular changes, consistent with our findings in both groups [32]. Demerdash et al. 2019, found telomere elongation in leukocytes post-DAA therapy in HCV-related cirrhosis, supporting our observation of altered telomere dynamics in the post-DAA group, likely due to treatment-induced molecular shifts [33]. Molina Carron et al. 2020, found similar finding in HCV eradication by oral DAA was associated with an increase in telomere length with advanced cirrhosis and suggested that DAA treatment may fundamentally alter molecular mechanisms underlying HCC development [31]. Moreover, Ningarhari et al. 2021, noted that telomere length elongation is more pronounced in HBV-related HCC due to etiology-specific telomerase mutations which occurs is about 70% in HCV versus 29% in HBV, such finding could explain the moderated elongation in our post-DAA cohort [34]. Correlation analysis of RTL with tumor markers AFP, CEA and CA19-9 revealed, non-significant correlations with tumor markers in the non-DAA group however, a significant correlation between tumor RTL and CEA in the post-DAA group was observed and might implies that DAA therapy may fundamentally alter the molecular landscape of HCC and other gastrointestinal carcinogenesis. The positive correlation suggests that in patients previously treated with DAA, changes in telomere length are more closely linked to CEA levels, potentially indicating a unique molecular adaptation mechanism. These findings underscore the complexity of hepatocarcinogenesis, particularly in the context of HCV infection and antiviral treatment. These differential correlation patterns were observed and partially supported by Loukopoulou et al. 2024, who linked telomere length to gastrointestinal cancer biomarkers, including CEA, suggesting shared molecular pathways which may reflect underlying molecular changes induced by DAA therapy, and potentially affecting telomere dynamics and GIT tumor marker expression [35]. In contrast, earlier study by Raynaud et al. 2008 found telomere shortening, not elongation, correlated with colorectal cancer progression, indicating our CEA-telomere association may be HCC-specific or DAA-driven, highlighting the need for further studies on treatment effects [36].

Clinical parameters provided crucial insights into the potential predictive value of telomere length. Significant variations in pathological staging and Child-Pugh classification between groups indicate that telomere length may reflect more than cellular aging, it could potentially serve as a comprehensive marker of hepatic health and cancer risk [37–39]. In the non-DAA group, the relationship between telomere length and clinical features demonstrated intricate patterns. Estimated tumor volume exhibited a striking negative correlation with telomere length, suggesting that lower telomere length was associated with larger tumor volumes. Histological grade showed a notable positive correlation, indicating that higher telomere length was associated with more aggressive tumor characteristics. Age displayed a moderate positive association, suggesting a subtle relationship between increasing age and telomere length variations. Conversely, the post-DAA HCC group revealed markedly different molecular dynamics. Estimated tumor volume demonstrated a positive correlation with telomere length. This stands in contrast to the non-DAA group, suggesting potential treatment-induced alterations in tumor biology. Child-Pugh classification exhibited a significant positive association, indicating that telomere length may be linked to liver functional status in patients who received antiviral treatment. Histological grade showed a modest positive correlation, further highlighting the nuanced molecular changes in this patient group.

These clinicopathological differences, such as aggressive tumor grades in the non-DAA group, and better liver function post-DAA, align with Ochi et al. 2021 who reported improved HCC outcomes post-DAA, likely due to enhanced liver function, supporting our findings [39]. However, Gellert-Kristensen et al. 2024, found no clear link between telomere length and liver function in a broader liver disease cohort, suggesting our Child-Pugh findings may be specific to HCV-related HCC post-DAA [40]. These findings underscore the complex and group-specific nature of telomere length dynamics in HCC. The divergent correlations between telomere length and clinical-pathological features in de novo and post-DAA patients suggest that antiviral treatment may fundamentally alter molecular mechanisms underlying HCC development. The variations in tumor volume, histological grade, and liver function associations point to potentially distinct pathogenic processes in these two patient populations. Despite these molecular and clinical insights, no significant association was found between RTL and HCC risk in either group. This aligns with Zeng et al. 2017, who reported no significant association between peripheral blood leukocyte telomere length and HCC risk in a Taiwan HCV cohort [41]. Yang et al. 2023, in a Mendelian randomization study also found no significant association between telomere length and HCC risk in Asian and European populations, reinforcing our findings that TL may not consistently predict HCC risk, and suggesting telomere length’s role may be context-dependent [42]. In contrast, Wan et al. 2017, reported a significant association between serum RTL and increased HCC risk in HBV patients. Differences in etiology (HBV vs. HCV), RTL measurement method, and cohort size likely explain this disagreement with our results [43]. On the other hand, some studies provide mixed insights, partially aligning with our findings but highlighting contextual differences. A meta-analysis by Zhang 2015, included 33 studies on TL and cancer survival/progression, including HCC found shorter leukocyte TL was associated with worse HCC survival, not risk, partially aligning with our null risk findings but suggesting TL’s prognostic role, which our study did not explore [44].

The innovative aspects of this study lie in its direct comparison of RTL dynamics between de novo (non-DAA) and post-DAA HCC, revealing DAA-specific molecular adaptations. In the non-DAA group, pronounced RTL elongation alongside aggressive Grade III predominance suggests persistent inflammation drives telomerase reactivation, accelerating chromosomal instability [34]. Conversely, post-DAA patients exhibited moderated elongation and better Child-Pugh scores, potentially reflecting reduced oxidative stress post-viral clearance, which slows telomere erosion but may unmask alternative pathways [28]. This heterogeneity underscores DAA’s dual role: extenuating liver damage while possibly altering tumor biology, as evidenced by the novel RTL-CEA correlation, implying shared epithelial-mesenchymal pathways in post-treatment surveillance [35]. Underlying mechanisms warrant exploration, such as TERT promoter mutations (prevalent in 70% HCV-HCC) or epigenetic shifts post-DAA [13, 31]; targeted sequencing could elucidate these. All post-DAA patients received sofosbuvir-based regimens (standard 12-week duration), but variability in treatment timing post-SVR may confound dynamics, meriting adjustment in future models [28].

Clinically, these findings suggest RTL as a marker for HCC heterogeneity, aiding risk stratification in post-DAA follow-up. For instance, elevated tumor RTL in non-DAA cases may signal higher recurrence risk, aligning with improved post-DAA outcomes [39]. However, ROC analysis indicates limited standalone discriminatory power, highlighting the need for future optimizations in telomere length measurement—such as terminal restriction fragment (TRF)-Southern blot for absolute quantification to minimize qPCR biases like primer dimerization—and multi-biomarker panels (e.g., RTL combined with AFP/CEA or circulating miRNAs) to improve prediction accuracy, as supported by recent reviews on telomere-targeted diagnostics [45, 46]. To further ensure authenticity in RTL quantification from FFPE tissues, which may retain minor non-tumor cell contamination despite >80% purity confirmation via H&E, future studies could employ laser capture microdissection for precise tumor cell isolation. Relationships with survival and recurrence remain unassessed here but are critical; meta-analyses link shorter RTL to worse HCC prognosis, supporting RTL’s potential beyond risk prediction [44].

### Limitations of the study

Despite the insights gained, several limitations must be acknowledged. The small sample size increases the risk of type II errors and limits statistical power for subgroup analyses, while the single-center Egyptian cohort restricts generalizability to other ethnicities and geographies. This modest sample size reflects inherent challenges in procuring HCC tissue samples from a single center, as diagnostic liver biopsies are increasingly avoided due to risks of tumor seeding and procedural complications associated with fine-needle aspiration. Contemporary guidelines favor non-invasive imaging modalities and reserve surgical resection for limited therapeutic contexts [47]. Additional constraints include the retrospective design, which limits uniformity in viral load measurements and introduces variability in DAA treatment duration as potential confounders; the use of synthetic controls from ANT tissues, which do not represent true healthy baselines; the absence of raw survival and recurrence data, precluding prognostic assessments; and the reliance on formalin-fixed paraffin-embedded (FFPE)-based RTL measurements, which may be influenced by fixation artifacts despite confirmation of >80% tumor purity via hematoxylin and eosin (H&E) staining. Furthermore, no longitudinal follow-up data were available, and the quantitative polymerase chain reaction (qPCR) method measures relative (rather than absolute) telomere length. These factors temper the conclusions, underscoring the exploratory nature of the findings. Future multi-center studies with larger cohorts, absolute telomere length measurement methods (e.g., terminal restriction fragment analysis), and inclusion of survival endpoints are essential to validate RTL’s prognostic utility and further dissect DAA mechanisms for personalized HCC management [34, 40, 45, 46].

## Conclusion

This study offers compelling insights into RTL elongation, carcinoembryonic antigen (CEA) correlations, and clinicopathological distinctions in HCV-associated HCC following DAA therapy. In clinical practice, tumor RTL—assessed from resection specimens in early-stage (BCLC 0/A) [47] post-DAA patients undergoing curative surgery—could stratify those with marked elongation (>1.5-fold vs. adjacent non-tumorous tissue) into higher-risk cohorts for intensified postoperative imaging (contrast-enhanced MRI every 3–6 months) and consideration of adjuvant therapies, thereby guiding personalized follow-up in this surgically managed subgroup.

## Data Availability

The datasets generated and/or analyzed during the current study are not publicly available due institutional policies but are available from the corresponding author on reasonable request.
